# Combined Ultrasound/Microwave Chemocatalytic Method for Selective Conversion of Cellulose into Lactic Acid

**DOI:** 10.1038/s41598-019-55487-y

**Published:** 2019-12-11

**Authors:** Sofia Tallarico, Paola Costanzo, Sonia Bonacci, Anastasia Macario, Maria Luisa Di Gioia, Monica Nardi, Antonio Procopio, Manuela Oliverio

**Affiliations:** 1 Dipartimento di Scienze della Salute, Università Magna Graecia, Viale Europa, 88100 Germaneto (CZ), Italy; 2 Dipartimento di Chimica, Università della Calabria, Cubo XXX, 87036 Arcavacata di Rende (CS), Italy; 3 Dipartimento di Farmacia e Scienze della Salute e della Nutrizione, Università della Calabria, Edificio Polifunzionale, 87036 Arcavacata di Rende (CS), Italy

**Keywords:** Environmental chemistry, Green chemistry

## Abstract

Cellulose is the main component of lignocellulosic biomass. Its direct chemocatalytic conversion into lactic acid (LA), a powerful biobased chemical platform, represents an important, and more easily scalable alternative to the fermentative way. In this paper, we present the selective hydrothermal conversion of cellulose and simple sugars into LA, under mild reaction conditions in presence of ErCl_3_ grafted on the mesoporous silica (MCM-41) surface. High yields and selectivity were obtained for the conversion of sugars under microwave (MW) irradiation at a relatively low temperature (200 °C) and short reaction times (10 min) under microwave (MW) irradiation. Ultrasounds (US) pre-treatment was investigated to reduce the cellulose crystallinity, before the MW-assisted conversion, providing LA with a yield of 64% within 90 min at 220 °C below the subcritical water conditions with increased operational safety. We finally discuss the scalability of the process and the recyclability of the catalyst.

## Introduction

Lactic acid (LA) is a chiral carboxylic acid traditionally used in the food industry as acidulant, preservative and emulgator^1^. It was recently added in a report of the US Department of Energy (DOE), describing the new platform chemicals deriving from biomass, which due to its wide reactivity allow the conversion, with similar technologies, into different products such as lactates, pyruvic acid, 2,3-pentandione, acetaldehyde, acrylic acids and their polymers, by using similar technologies^2^. Furthermore, LA itself, is used as monomer for the synthesis of biodegradable polymers such as polylactic acid (PLA), which has a wide range of applications, from pharmaceutics to personal care products and packaging materials^3^. PLA, with its low cost and availability on the market, is the first polymer from biodegradable resources to have the chance to replace petroleum-based polymers in electronic, agricultural, textile and other market segments^4^.

LA is traditionally produced by fermentation of trioses and hexoses coming from lignocellulose biomass, but this route suffers from a sustainability drawback^5^. Which is why the actual production of LA, even though continuously growing, is still lower compared to the commodity demand, thus justifying the exploration of new chemocatalytic routes to obtain LA directly from lignocellulose biomass.

Chemocatalytic conversion of cellulose has been recently explored as a sustainable alternative for LA production, although its selective transformation is still a hard issue^6^. Indeed, cellulose has a recalcitrant nature, needing hard operative conditions in terms of temperatures and pressures to activate its solubilization and degradation in water. The hydrothermal degradation, not only useful for conversion into add-value chemicals of crystalline, but also for lignocellulosic materials^7^, and pure monosaccharides^8^, can be easily realized using microwave heating^9–11^. When MW-assisted hydrothermal degradation of cellulose is performed without any catalyst, temperatures above 240 °C are needed to activate cellulose decrystallinization, depolymerization and hydrolysis, while only the amorphous portion of cellulose reacts at lower temperatures^10^.

Several different homogeneous^12–14^ and heterogeneous^15–18^ acid and base catalysts have been tested under sub-critical water conditions (190 < T (°C) < 280, 217 < P (psi) < 440) with LA yields ranging from 22% to 68%^3^.

Although, good results were obtained at relatively low temperatures using lead(II) ions^12^, the high toxicity of the metal and the necessary long cellulose pre-treatment needed to reduce the crystallinity (obtained by ball-milling the microcrystalline cellulose), make this protocol not entirely green.

Interestingly, the best conversion performances have been reached using Er(III) salts in aqueous solution, both in the homogeneous and heterogeneous phase^14,16,18^, at 240 °C under 290 psi of N_2_ pressure in autoclave.

Looking for a heterogeneous, effective route to apply Er(III) catalysis to the LA selective production, two heterogeneous Er(III) catalysts were proposed: ErCl_3_ adsorbed on K10 montmorillonite and ErCl_3_ grafted on β-zeolite. In both cases the conversions approached the results obtained in the homogeneous phase^14,16^, but the transformations suffered from the common drawback of failing the recycling procedures, due to the formation of a huge amount of carbon black residues poisoning the heterogeneous catalysts^13,18^. Such residues were mainly due to the hard reaction conditions needed to activate cellulose de-crystallization, solubilization and hydrothermal degradation.

Regarding the importance of the crystallinity on the cellulose hydrothermal hydrolysis, the effect of acoustic cavitation on water suspensions of lignocellulosic biomass by US exposition was recently studied, and the ability of US to disorganize the crystalline lattice of cellulose was demonstrated^19–21^.

Homogeneous and heterogeneous Er(III) salts catalysis was extensively studied by our research group^22^ under non-conventional reaction conditions such as microwaves (MW)^23–26^ and ultrasound (US)^27^ heating, as well as soft pressurized^28^ and continuous flow^29^ reactors.

The aim of this study is to propose a catalytic green alternative to selectively realize direct cellulose conversion into LA, lowering the energy demand, the overall complexity and the time of the process in order to achieve a realistic scaling-up procedure.

In this paper, we present an alternative US/MW combined method to realize the selective hydrothermal conversion of hexoses and cellulose into LA, under mild reaction conditions in presence of ErCl_3_ grafted on MCM-41 silica surface. We demonstrated that, because of the effect of US, its MW-assisted conversion into LA was possible at temperature and pressure below the subcritical water conditions. A critical approach to the use of Er(III) based heterogeneous catalysts is also proposed. Indeed, concerning the catalyst recovery and reuse, we definitively disclosed that the production of carbon residues is not the main reason of recycling failures, while the erbium leaching remains an important drawback. Finally, a scale up of the process has been realized for both fructose and cellulose as starting materials.

## Experimental Section

### Instruments

The reactions performed in the high energy density laboratory CEM Discover Microwave were run on dynamic mode, applying a specified amount of power to reach the desired temperature and pressure, working with borosilicate glass vessels equipped with silicon cap with septum.

Hydrothermal reactions in the multiwave oven were carried out using a Synthos 3000 (Anton Paar) equipped with a rotor XF100 with PTFE-TFM vessels, operating at a magnetron frequency of 2455 MHz.

The ultrasound-assisted pre-treatment of microcrystalline cellulose (MCC) was performed using a 20,7 Hz high power US system equipped with a titanium immersion horn made by Danacamerini s.a.s.

Sample analyses were performed on a HPLC Thermo Scientific Dionex Ultimate 3000, equipped with a 250 × 4.6 mm Thermo Scientific Hypersil GOLD C18 column packed with 5 μm particles. For the HPLC separation of the products, a gradient elution with a mixture of solvents A (H_2_O+ phosphoric acid, pH = 2,20) and B (acetonitrile) was used. The column was equilibrated in 95% solvent A and 5% solvent B. The elution flow rate was 1 ml min-1 by linearly increasing the solvent B concentration from 5 to 75% in 17 min, then return to 5% in 10 min and equilibrated in 3 min. The chromatograms were acquired at four different wavelengths: 210 nm for lactic acid, 266 nm for levulinic acid, 278 nm for furfural and 284 nm for 5-HMF.

The instrumentation performance, chromatograms, and initial data processing were carried out with Chromeleon software. Calibration curves were built using standard solutions of each compound.

The quantification of saccharides was determined by a HPLC Agilent series 1100 system coupled with a refraction index detector (RID G1362A), equipped with a Luna-NH_2_ column (250 × 4.6 mm, particle size 5 µm), working at 35 °C, using an eluent mixture of acetonitrile/Milli-Q water 75:25 and a flow rate of 2 ml min-1.

Qualitative analyses were performed using electrospray ionization mass spectrometry (ESI-MS). A 6500 QTRAP Mass spectrometer (AB Sciex) was used. The QTRAP-MS system was equipped with an electrospray ionization source (ESI), operated in negative and curtain positive ion mode.

ESI worked under the following conditions: gas at 20 psi, nebulizer gas at 50 psi, ionization mode source voltage −4500 V. Nitrogen was used as curtain and collision gas. The data were acquired and processed using Analyst 1.5 software.

The total organic content (TOC) of cellulose reactions was determined by using an Analytik Jena AG TOC Analyzer multi N/C 2100.

### Materials

D-(-)-fructose (≥99%), D-(+)-glucose (≥99%), D-(+)-cellobiose (≥99%), MCC (particle size 51 µm), DL-lactic acid (certified reference material), levulinic acid (≥99%), 5-(hydroxymethyl)furfural (≥99%) and furfural (analytical standard, purity ≥97,5%) were purchased from Sigma Aldrich. Er^III^-MCM-41 was synthetized using the previously reported procedure^30^. Both the acetonitrile and phosphoric acid used in HPLC were of analytical grade (Sigma Aldrich) and used without further purification.

### Hydrothermal degradation procedure of hexoses

For preliminary experiments concerning simple sugars, 0,1 g of substrate, 0,03 g of catalyst and 10 mL of water were poured into the vessel and put in position 1 of the XF100 S8 rotor (Synthos 3000, Anton Paar), equipped with a T-probe temperature sensor to monitor the reaction. Position 3, 5 and 7 were occupied by vessels filled with water to balance the symmetry of the rotor. For these studies, reaction temperatures of 180 and 200 °C and times ranging from 5 to 30 minutes were investigated. A pre-heating ramp of 5 minutes was applied for all reactions and the maximum reached power was 1000 W.

The final solution was transferred in a vial, filtered and diluted prior to analysis in Milli-Q water. Conversion, yields and selectivity were determined by HPLC analysis.

Concerning the scalability of the process, all eight positions of the rotor were employed, the quantities were proportionally incremented by a factor 3 and the maximum power was set at 1200 W in order to ensure the reproducibility of the reaction on all vessels.

### Hydrothermal degradation procedure of MCC

MCC (0,1 g) was placed in a flask with 30 mL of water and pre-treated before the microwave reaction, using an ultrasonic immersion probe for direct sonication for an hour (power 10 W, amplitude 25%). The obtained solution was transferred to the vessel and 0,03 g of catalyst was added. Consequently, the reaction was performed at different temperatures between 200 and 220 °C for several time ranges.

According to HPLC and TOC analysis, the product yields and the conversion of the reaction were calculated as follows:

## Results and Discussion

Starting with the data reported in the literature we decided to study: (i) the best MW-assisted conditions to perform the conversion of simple hexoses into LA and (ii) the coupling of MW heating with US cellulose pre-treatment for its selective conversion into LA. We selected a hybrid, heterogeneous, high superficial area mesoporous catalyst, previously synthetized by grafting ErCl_3_ onto the MCM-41 silica surface (Er^III^-MCM-41)^15^. Such catalyst was already used to efficiently realize a chemocatalytic transformation of small molecules under non-conventional media^23–29^ and mild reaction conditions.

### MW-assisted conversion of hexoses into LA

In order to find the best MW reaction conditions for hexoses and disaccharides, we performed the hydrothermal degradation of fructose, glucose and cellobiose by using a mesoporous ErIII-MCM-41 catalyst, and carrying out the reaction at temperatures ranging from 180° to 200 °C, screening different intervals of time as reported in Table 1.

**Table 1:** Chemo-catalityc MW-assisted conversion of hexoses and disaccharides into LA.

Entry	Substrate	T (°C)	t (min)	p (psi)	Conv.^a^ (%)	Yield ^b^ (%)				Sel. in LA (%)
						LA	5-HMF	Furfural	Levulinic acid	
1^c^	Fructose	180	3	180	72	44	5	0,5	n.d ^d^	89
2^c^		180	10	180	75	58	11	0,6	0,8	82
3^c^		180	30	180	87	73	12	1	1	84
4^e^		200	5	200	90	78	13	0,8	n.d	85
5^e^		200	10	200	90	73	13	0,8	n.d	84
6^c^		200	15	200	87	77	12	0,8	n.d	86
7^c^		200	20	200	84	66	2	0,9	n.d	96
8^c^		200	30	200	89	73	7	0,7	n.d	90
9^e^	Glucose	200	5	200	75	45	12	0,7	n.d	78
10^e^		200	10	200	78	59	13	1	n.d	81
11^e^	Cellobiose	200	5	240	75	57	11	1	n.d	82
12^e^		200	10	275	95	75	14	1	n.d.	83

^a^Reaction conditions: 0,100 g of substrate, 0,030 g of ErIII-MCM-41 (13,4 wt%) and 10 mL of MilliQ water; conversion calculated by HPLC-RI analysis. ^b^Yield calculated by HPLC-UV. ^c^Reaction performed in CEM Discover Microwave reactor. ^d^Concentration <0,5%. ^e^ Reaction carried in Synthos 3000 Anton Paar Microwave reactor.

The concentration of the sugar solutions and the amount of catalyst were chosen based on the data reported in the literature^13,18^. Performances of two different MW ovens, namely monowave CEM Discover Microwave reactor and multiwave Synthos 3000 Anton Paar Microwave reactor, were compared. Conversions and yields of representative products (>0,5%) were respectively evaluated by HPLC-RI and HPLC-UV analysis of the reaction mixture. Quantification was performed after a comparison with calibration curves of the respective standards. As assumed from Lei *et al*.^14^, the degradation of sugars in the presence of a Lewis acid could follow two possible pathways starting with the inter-conversion of glucose into fructose:^9^ one involved the dehydration of fructose leading to the formation of levulinic acid and formic acid; the other implicated the formation of trioses which converted into LA.

As expected from the mechanistic data reported in the literature^13,14,18^, LA was the major product for all tested substrates, with the formation of on average 10% of 5-HMF and very small amounts of furfural and levulinic acid, which in some cases were completely absent.

At first, the reaction of fructose was performed in a high energy density laboratory CEM Discover Microwave, running on dynamic mode, working with a power of 200 W to reach the desired temperature and pressure (entries 1–3, Table 1). Then, we decided to repeat the reaction at 200 °C in the reactor Synthos 3000, which allows to achieve and maintain medium-high temperatures and pressures, in a safer manner compared to a classic autoclave. Better performances in terms of reaction time, yield and selectivity were recorded (entry 4, Table 1). Indeed, we were able to convert 90% of fructose in only 5 minutes at 200 °C, thus obtaining lactic acid with a yield of 78% and a selectivity of 85%. Longer reaction times did not improve the reaction yield and selectivity (entries 5–8, Table 1) despite the diminution of 5-HMF and the absence of levulinic acid; on the contrary, an increase of deposition of carbon species was noticed. Furthermore, a qualitative analysis of the reaction by direct infusion ESI mass spectrometry (See Fig. S1, Electronic Supplementary Materials) disclosed that extended reaction times promoted the formation of the LA dimer.

Moreover, Er^III^-MCM-41 chemocatalytic routes allowed a glucose conversion to LA (entry 10, Table 1) in 10 minutes with good yields (59%) and selectivity (81%), despite its natural rigidity^1^.

Conversion of cellobiose has been similar to that of the glucose under the same working pressures (entry 11, Table 1), while at slighty higher operating pressures allowed to reach 75% of LA yield with a selectivity of 83% (entry 12, Table 1) thus confirming pressure itself as a pivotal parameter in these kinds of conversions. Again, in both glucose and cellobiose conversion, longer times did not correspond to better performances (data not showed).

**Figure 1:**
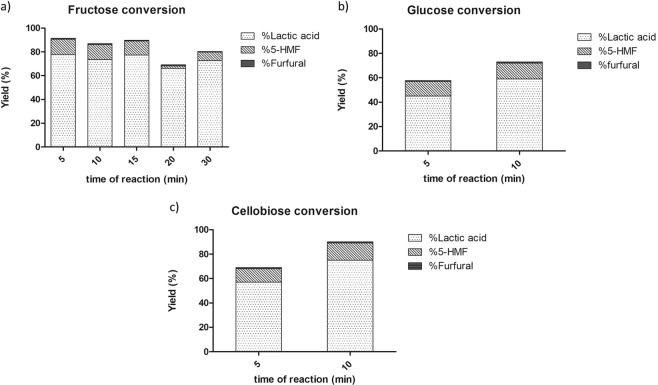
Effect of reaction time on conversion of (**a**) fructose, (**b**) glucose and (**c**) cellobiose into LA.

Figure 1 graphically describes the results in terms of yields and selectivity at different times for all of the tested substrates.

As a matter of fact, we were able to realize a selective conversion to LA of all substrates, whit a ranging yield of 57% to 78% and a selectivity up to 85%, in relatively mild and safe operational conditions compared to classical autoclaves, thus demonstrating the efficiency of the proposed chemocatalytic MW-assisted route. The formation of carbon residues was observed in all reactions, but we noticed a strict direct correlation with the reaction time. The reaction mixes obtained after 5–10 minutes were clearer than the ones with longer reaction times.

### Combined US/MW-assisted conversion of MCC into LA

MCC is a purified cellulose prepared by treating alpha-cellulose, which is obtained as pulp from fibrous plant material. It is known from the literature that cellulose only reacts to hydrothermal degradation with its amorphous region, especially at temperatures beneath 220 °C^10^.

For this reason, we decided to introduce a preliminary step of cellulose pre-treatment, thus to preserving the mild conditions obtained for the hexoses and cellobiose. Ultrasonic energy,capable of decreasing the recalcitrance of cellulose and enhancing the processes of depolymerisation and decrystallisation, can meet this challenge^31,32^. An ultrasonic probe system (Danacamerini s.a.s., 20,7 kHz, amplitude 25%) was employed for direct pre-sonication of a water suspension of cellulose, at different powers and times.

US pre-treated cellulose, at all tested powers, was then reacted under MW-assistance at different temperatures and times as schematically reported in Table 2.

**Table 2:** Summary of the hydrothermal reactions of cellulose^a^.

Entry	MW T (°C)	Internal P (psi)	t (min)	US Power (W)	Conv.^b^ US/no US	Yield^c^ of LA US/no US	Sel. US/no US
1	200	390	60	10 (60′)	10/8	4,8/3,8	93/82
2	200	390	90	10 (60′)	38/10	20,6/4,6	92/87
3	200	390	120	10 (60′)	59/13	32,1/5,9	86/83
4	200	390	120	10 (120′)	8/13	n.d. /5,9	—/83
5	200	390	120	20 (60′)	7/13	n.d. /5,9	—/83
6	200	390	120	50 (60′)	8/13	n.d. /5,9	—/83
7	200	390	120	100 (60′)	5/13	n.d. /5,9	—/83
8	210	420	60	10 (60′)	56/10	31,4/4,8	97/86
9	210	420	90	10 (60′)	64/ 12	34,2/5,2	90/87
10	220	490	60	10 (60′)	71/ 43	49,2/26,6	96/92
11	220	490	90	10 (60′)	88/67	63,9/35,5	94/90

^a^For all the experiments 0,100 g of MCC, 0,030 g of Er^III^-MCM-41 and 30 mL of MilliQ water. ^b^Conversion calculated by TOC analysis. ^c^Yield calculated from the analysis of crude reaction with HPLC-UV.

**Figure 2:**
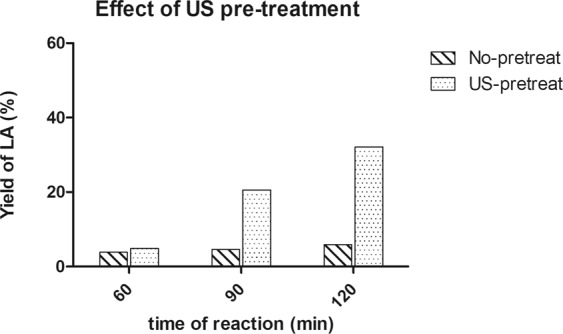
Effect of US pre-treatment on cellulose hydrothermal reaction at 200 °C in terms of Lactic acid yield.

The introduction of ultrasonic pre-treatment positively influences the reaction profile, with a significant increase of cellulose conversion, LA yield and selectivity after pre-treatments at 10 W (entries 2–3, 8–11, Table 2), compared to the reactions carried out without any initial pre-treatment. Surprisingly, a detrimental effect on reaction performances was registered upon increasing the US time exposure (entry 4, Table 2) or US power (entries 5–7, Table 2). In these cases, the conversion of cellulose into LA failed, and the HPLC profiles showed the appearance of a pronounced peak on the dead volume, probably composed by a complex mix of unknown compounds as shown by the ESI/MS analysis (see Figs. S3 and S4, Electronic Supplementary Materials). Therefore, we clearly demonstrated that mild US pre-treatment allowed performing selective cellulose conversion into LA at mild and safe conditions, while a more intense sonication drives the reaction to different pathways. Nevertheless, the reasons of such behavior need to be further explored. No significant improvements were registered when working at 210 °C (entries 8–9, Table 2), while, as expected, at subcritical reaction conditions (i.e. 220 °C and 490 psi) a yield of 64% was obtained after 90 minutes of reaction (entries 10–11, Table 2). LA selectivity was high in all examined cases (>90%). Figure 2 shows the performance improvement of the combined US/MW method at mild temperature conditions (T = 200 °C).

A slight amount of LA dimer was also present, as disclosed by ESI/MS analysis of the mixture (See Fig. S2, Electronic Supplementary Materials). According to the results obtained with simple sugars, the reaction time of MW-assisted hydrolysis did not seem to be a critical parameter to improve reaction yield, while it was responsible for an increasing of LA dimer. For such reason, longer reaction times were not investigated in cellulose conversion. In summary, the optimized reaction conditions for the combined US/MW mild hydrothermal degradation of cellulose were: US pre-treatment of cellulose for 60 minutes at 10 W Power, followed by an MW-assisted reaction at 200 °C for 120 minutes, thus obtaining LA with yield of 31% and 86% of selectivity

### Scale-up and catalyst recycling

To make the process more appealing on a large scale, we went on to demonstrate the scalability of the process by working with higher quantities of reactans. Thus, we decided to proportionally increase the quantities of substrate, catalyst and water and simultaneously to use all positions of the Synthos 3000 XF100 rotor (See Table S1, Electronic Supplementary Materials).

**Figure 3:**
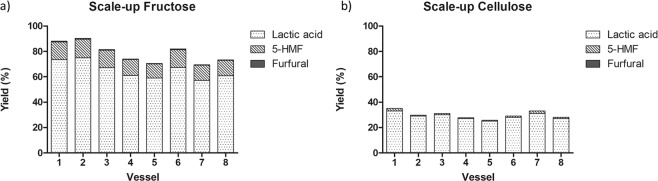
Scale-up of the fructose (panel a) and MCC (panel b) conversion into LA.

As shown in Fig. 3, reaction performed on fructose can be scaled up by reacting 2,4 gr of substrate in a single 10 min step, thus obtaining a LA yield of 65% and selectivity of 82%. Similar results were obtained with glucose and cellobiose (data not showed); concerning cellulose, after US pre-treatment of 2,4 gr of MCC at 10 W for 1 hour, followed by MW heating for 2 hours at 200 °C, it was possible to obtain a 30% average yield of LA with an average selectivity of 96%.

**Figure 4:**
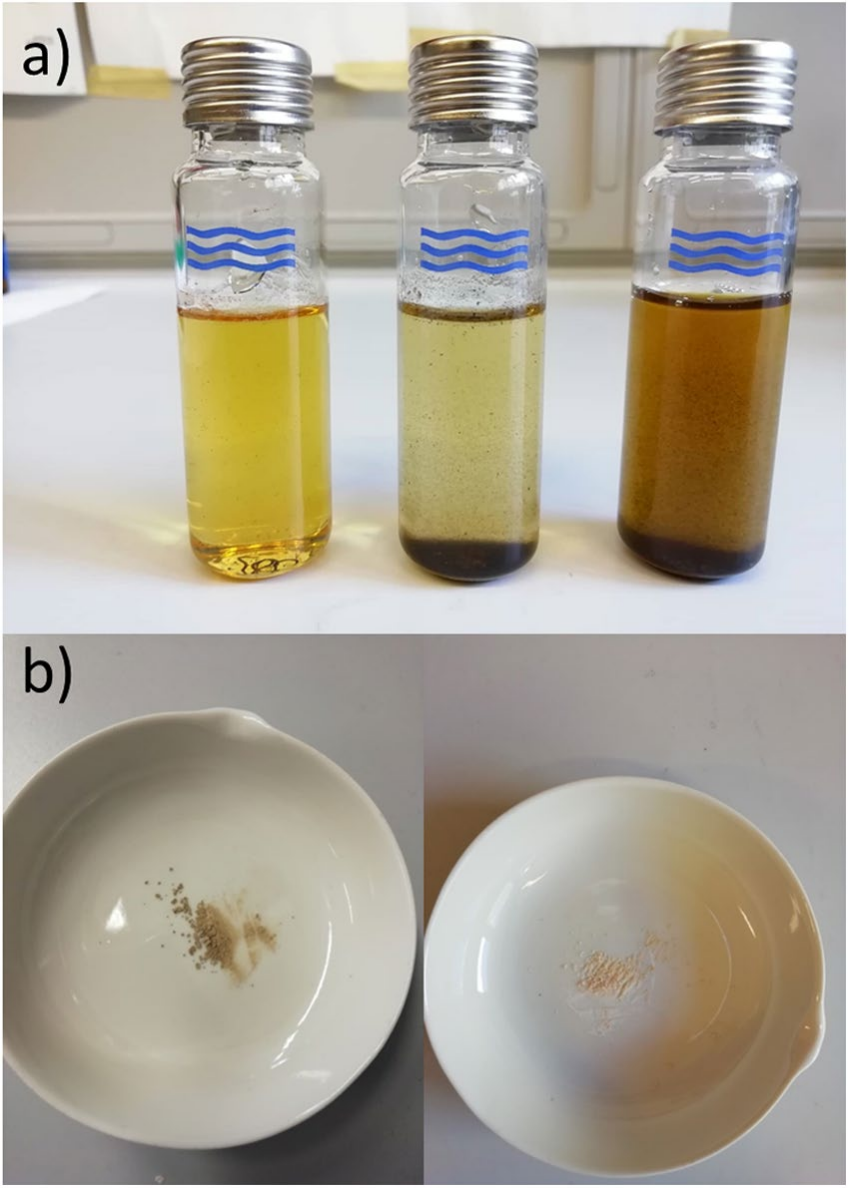
(**a**) Reaction mix gained at 200, 210 and 220 °C (reaction time of 120, 90 and 90 minutes, respectively). In panel b, the effect of calcination in order to remove carbon residues on our catalyst (on the left the catalyst after filtration and washing; on the right the catalyst after calcination process).

Finally, the recyclability and reusability of Er^III^-MCM-41 was evaluated. For simplicity, we evaluated the recycling of the catalyst at the best reaction conditions of fructose and cellulose conversion. As expected, the milder reaction conditions of the MW-assisted method allowed to lower the production of solid carbon residues, which proportionally increased with increasing temperatures (Fig. 4, panel a). After each run, the catalyst was separated from the reaction environment by filtering it on a fritted glass filter, washed with MilliQ water, dried at 100 °C and calcinated at 400 °C for 3 hours in a static air oven. Due to the limited carbon residues absorbed, the calcination had a good fate, thus restoring the total surface area of the catalyst (Fig. 4, panel b). Such data were confirmed by a porosimetric analysis performed on the catalyst before and after the reaction and calcination (See Table S2, Electronic Supplementary Materials). Nevertheless, it is worth to note that a change in total BET and in pore diameter was registered, thus suggesting that MCM-41 could not be perfectly resistant to such hard reaction conditions.

Moreover, a significant loss of LA yield was registered, thus reducing the catalyst performance by 50% after three reaction runs. As summarized in Table 3, after the calcination process, with each recycling trial a weight loss of the catalyst was observed due to the Er(III) leaching from its surface into the reaction solution.

**Table 3:** Catalyst recycling^a^.

Run	Erbium content (wt%)^b^	Conversion^c^	Yield^d^ (%)				Selectivity
			Lactic acid	5-HMF	Furfural	Levulinic acid	
1	13,4	90	74	14	0,6	n.d.^e^	84
2	9,1	84	52	13	0,5	n.d.^e^	79
3	0,5	70	31	13	0,3	n.d.^e^	70

^a^All reactions were performed with 0,300 g of fructose, 0,090 g of catalyst and 30 mL of MilliQ water. Reaction time of 10 minutes, temperature of 200 °C and pressure of 200 psi for all runs. ^b^Data obtained from ICP-MS analysis. ^c^Conversion calculated by HPLC-RI analysis. ^d^Yield of products calculated from the analysis of crude reaction with HPLC-UV. ^e^The concentration of the product from HPLC analysis led to yield lower than 0,5%.

Indeed, Erbium is has a very oxyphilic character^22^ and it can be guessed its higher affinity for the solution, rich in oxygenated molecules, rather that silica surface. In fact, even if quite all the Er(III) was lost from the surface still some conversion and yield can be registered at the third run, thus demonstrating a catalytic activity of Er(III) solved in the reaction mixture. ICP-MS data on the Er(III) content of reaction mixture after filtering off the catalyst, confirmed our hypothesis (See Table S3, Electronic Supplementary Materials). Moreover, in order to clarify if MW-irradiation could be responsible for such leaching, we performed a “blank” experiment, thus making the catalyst, react for two runs, only in presence of water and under both conventional and MW-assisted heating. After the filtration we compared the Erbium leaching between the two blank experiments two blank experiments and the MW-assisted hydrothermal degradation of fructose. As it was reported in Table S3, Electronic Supplementary Materials, the leaching data in the blank experiments were lower than those registered after hydrothermal degradation (64%), but still significant and similar between them (MW 24%; Classical 19%). Such results once again disclosed that the oxygenated character of both the solvent and the reaction mixture, was relevant for the metal leaching. On the contrary, no influence of MW-irradiation was observed. So, an important aspect to take into account in order to perform selective cellulose conversion into LA using heterogeneous catalysis is the oxophilicity of the metal center. Further studies are in progress to develop newly decorated silica surfaces able to graft Er(III) stronger than the oxygen rich mixture solution., are now in progress.

## Conclusions

In conclusion, we presented a combined US/MW-assisted method, catalyzed by MCM-41 Er(III) for the conversion of hexoses and cellulose into lactic acid. The method is efficient and selective for hexoses: MW assistance alone allowed mild reaction temperatures, safely operational conditions and good scaling-up performances. Concerning cellulose, an additional US pre-treatment at mild US power was necessary to activate its conversion. The US effect was clearly demonstrated to be “power selective” by our results, even if additional studies are needed to understand the reason of such an activation. Mild reaction conditions, namely under subcritical water temperatures, allowed to reduce carbon black residues normally produced during cellulose degradation processes, thus improving the catalyst calcination performance. Nevertheless, the oxyphilic character of Er(III) still influenced the metal leaching from silica surface, thus limiting the catalyst recycling. Further experiments selecting less oxyphilic lanthanides need to be performed.

## Supplementary information

Supplementary Information
