# Supplementary material for: Combined Ultrasound/Microwave Chemocatalytic Method for Selective Conversion of Cellulose into Lactic Acid
Source: Sci Rep. 2019 Dec 11;9:18858. doi: 10.1038/s41598-019-55487-y (PMC6906372; doi:10.1038/s41598-019-55487-y)
Supplement: Supplementary file 1 — Supplementary Information [file 41598_2019_55487_MOESM1_ESM.docx]

**Supplementary material**

**Combined Ultrasound/Microwave Chemocatalytic Method for Selective Conversion of Cellulose into Lactic Acid.**

Sofia Tallarico,^a^ Paola Costanzo,^a^ Sonia Bonacci,^a^ Anastasia Macario,^b^ Maria Luisa Di Gioia,^c^ Monica Nardi,^a^ Antonio Procopio,^a^ Manuela Oliverio.*^a^

^a^ Dipartimento di Scienze della Salute, Università Magna Graecia, Viale Europa, 88100-Germaneto (CZ), Italy

^b^ Dipartimento di Chimica, Università della Calabria, Cubo XXX, 87036-Arcavacata di Rende (CS), Italy

^c^ Dipartimento di Farmacia e Scienze della Salute e della Nutrizione, Università della Calabria, Edificio Polifunzionale, 87036-Arcavacata di Rende (CS), Italy

Direct infusion analysis by Mass Spectrometry 2

**Fig. S1** MS spectrum of Fructose reaction mix 3

**Fig. S2** MS spectrum of Cellulose reaction mix 4

Analysis of Cellulose reaction mix for 50 W US pre-treatment 5

**Fig. S3** HPLC chromatogram 5

**Fig. S4** MS spectrum of Cellulose reaction mix 5

Scaling-up of the process 6

**Table** **S1** summary of results gained in the trial for scalability of the process 6

Characterization of the catalyst after recycles 7

**Table** **S2** Characteristics of catalysts surface 7

**Fig.** **S5** Isotherm plot of fresh Er(III) MCM-41 catalyst 8

**Fig.** **S6** Pore volume distribution of fresh Er(III) MCM-41 catalyst 8

**Fig.** **S7** Isotherm plot of Er(III) MCM-41 catalyst after 2^nd^ run 9

**Fig.** **S8** Pore volume distribution of Er(III) MCM-41 catalyst after 2^nd^ run 9

ICP-MS determination of Er(III) during catalyst recycling 10

**Table** **S3** Er(III) content determination during catalyst recycling 10

**Direct infusion analysis by mass spectrometry**

Analysis of fructose and cellulose crude reactions were carried out by using electrospray ionization mass spectrometry (ESI-MS). The reaction mix was filtered by using a 0,45 μm PTFE filter and then diluted with MeOH (Methanol for HPLC-PLUS-Gradient, Carlo Erba Reagents) prior to the analysis in order to reach a concentration of 100 mgL^-1^. A 6500 QTRAP Mass spectrometer (AB Sciex, USA) was used. The QTRAP-MS system was equipped with an electrospray ionization source (ESI) operated in the negative ion mode.

ESI worked at the following conditions: curtain gas at 20 psi, nebulizer gas at 50 psi, ionization mode source voltage −4500 V, temperature of ion source 200°C, declustering potential -60 V, entrance potential -10 V. Nitrogen was used as curtain and collision gas. The data were acquired and processed by using Analyst 1.5 software (AB Sciex, USA).

**Figure S1**:*MS spectrum in negative ion mode of the crude reaction of fructose at 200°C for 10 minutes. In the panels the major products of the hydrothermal degradation are identified. The peak 96,9 is an interferent already present in the blank analysis.*


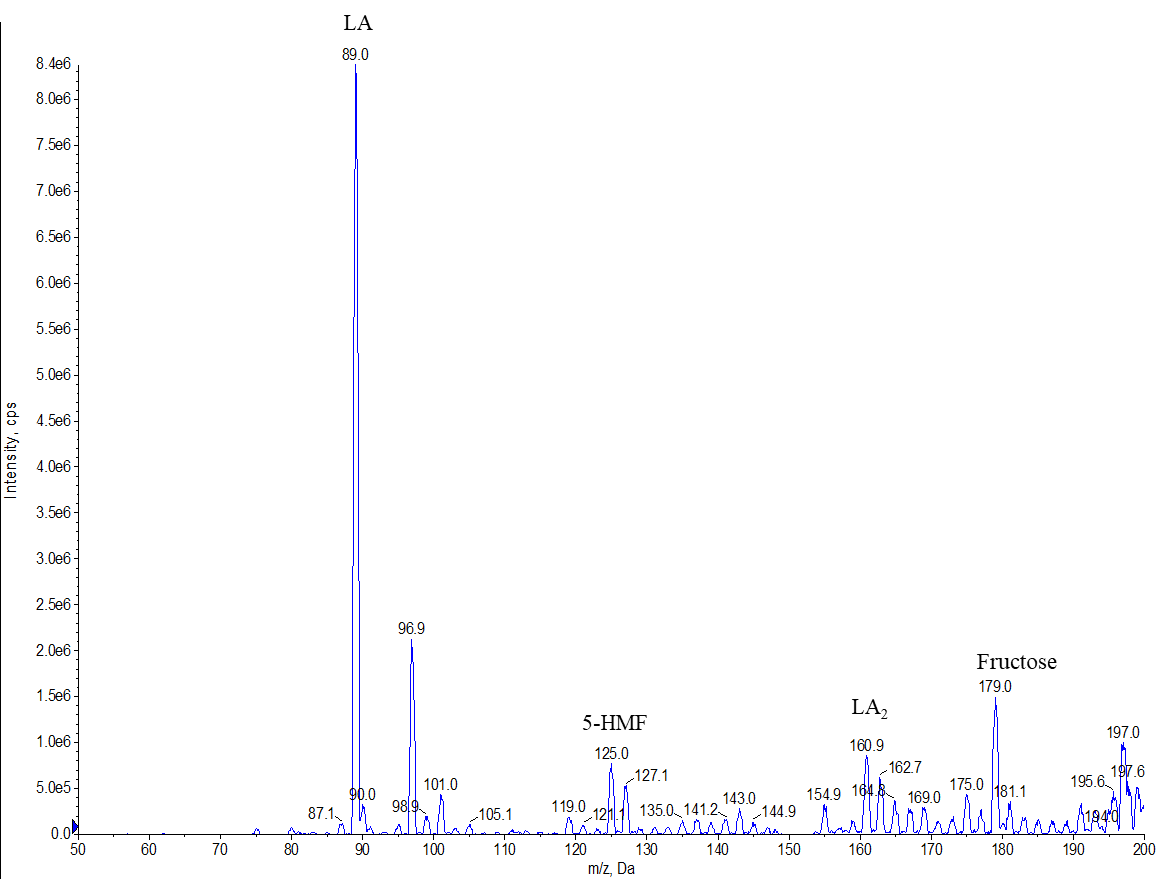


**Figure S2**:*MS spectrum in negative ion mode of the crude reaction of US pre-treated MCC at 200°C for 120 minutes. In the panels the major products of the hydrothermal degradation are identified. The peak 96,8 is an interferent already present in the blank analysis.*


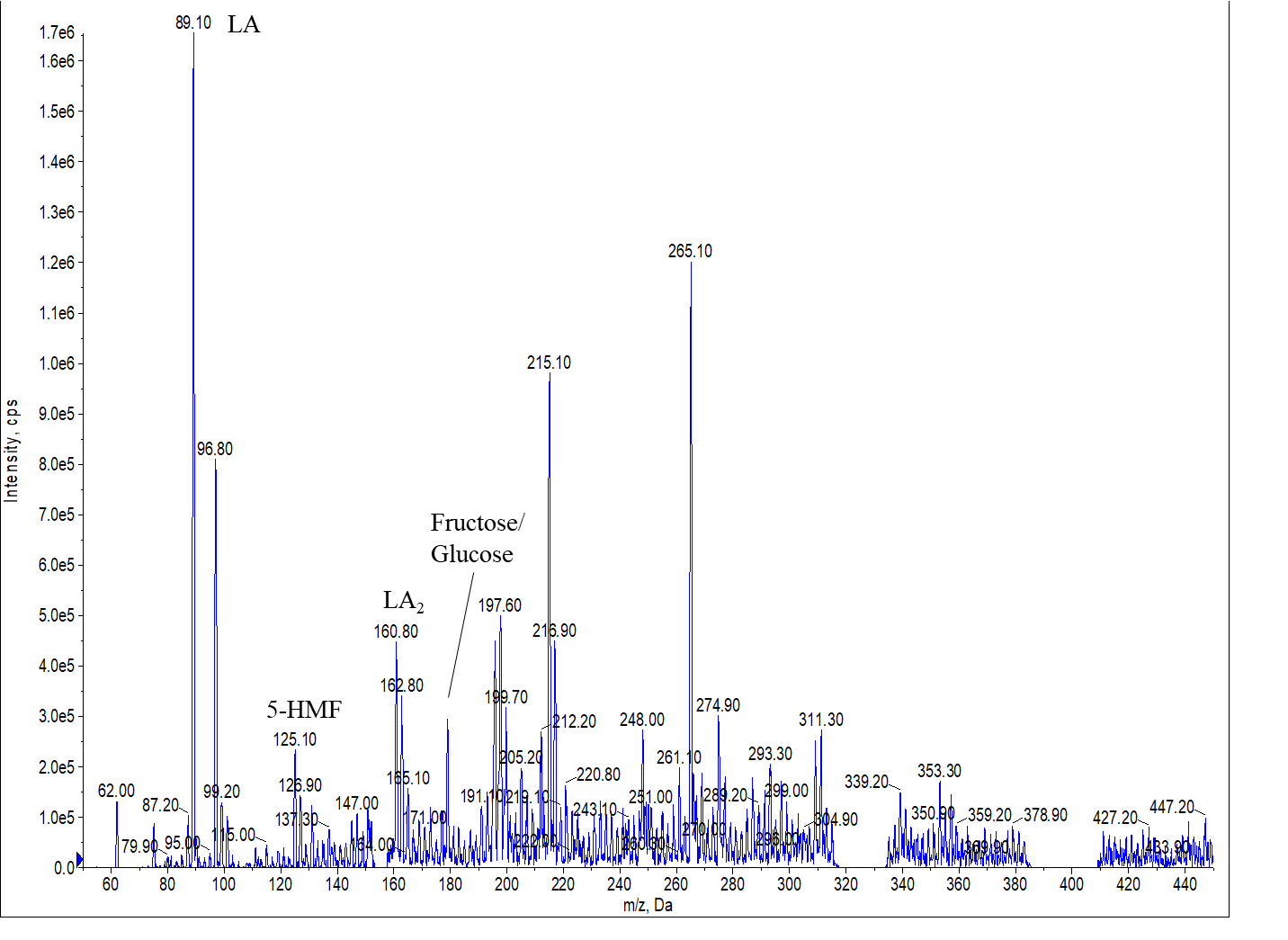


**Analysis of Cellulose reaction mix for 50 W US pre-treatment**

**Fig. S3** Comparison of the HPLC chromatogram of the crude reaction for US pre-treated cellulose at 50 W (in blue) and the chromatogram of the analytical standards for lactic acid, 5-HMF and furfural (in black) acquired at 210 nm.


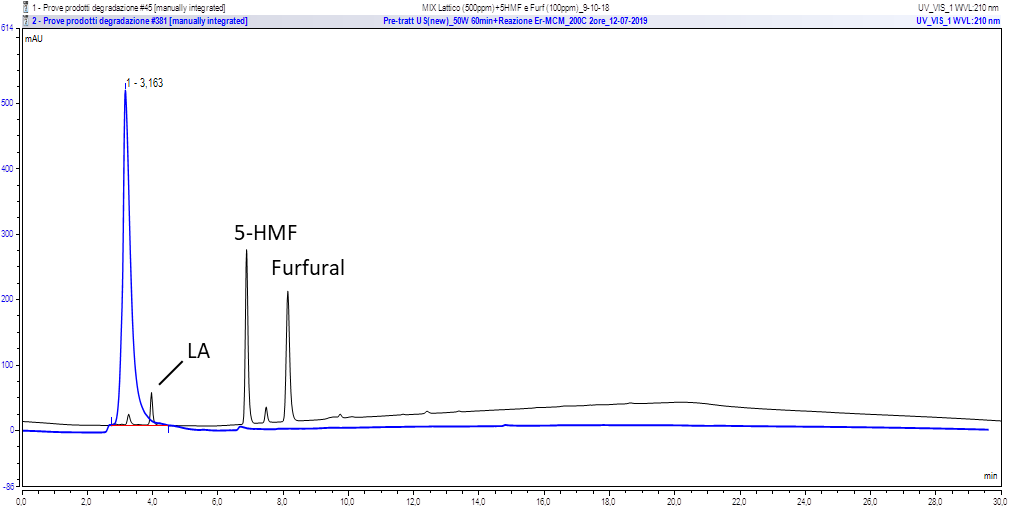


**Fig. S4** MS spectrum of Cellulose reaction mix**
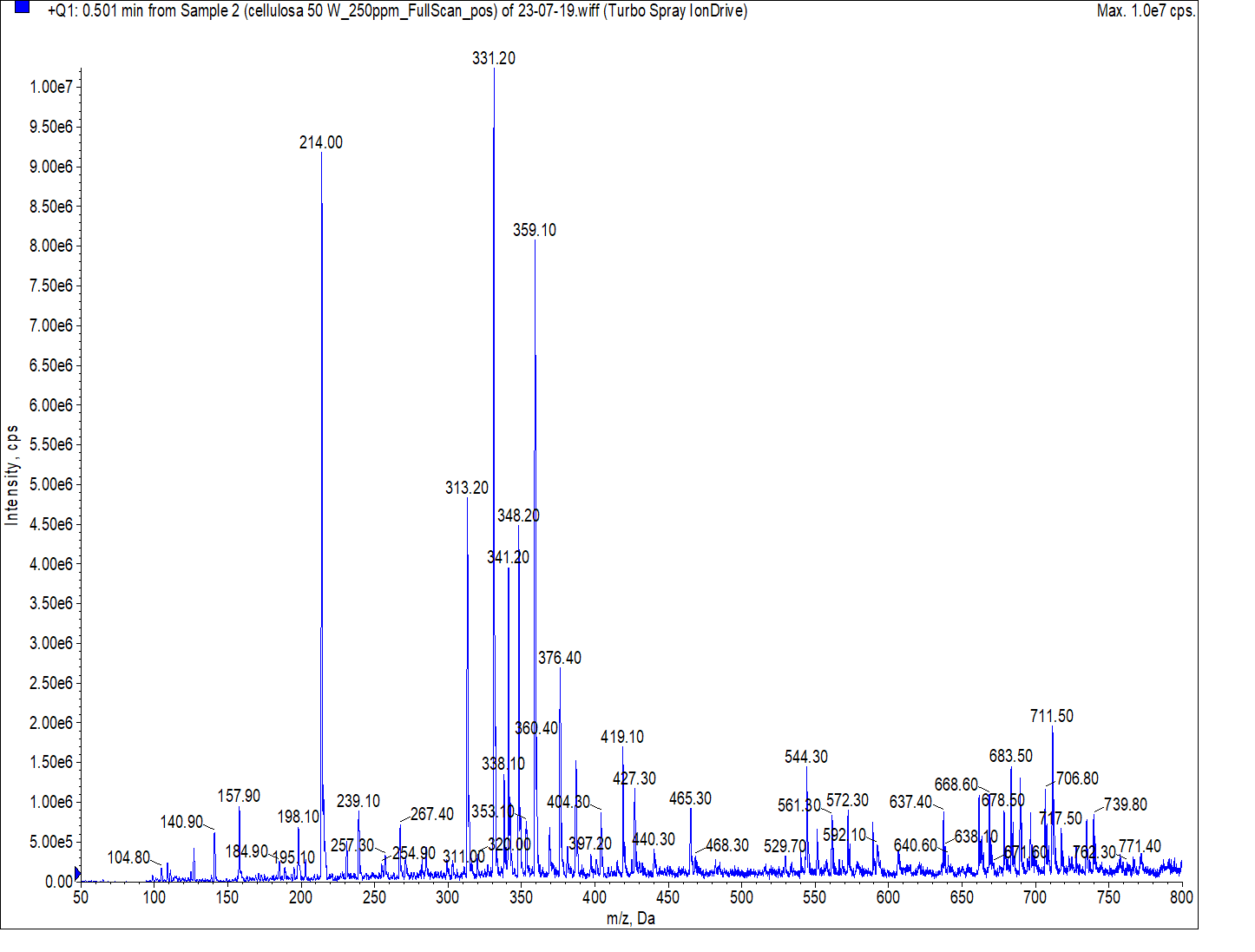
**

**Scaling-up of the process**

The hydrothermal reaction in each vessel was carried out at 200°C (power 1200 W) with 0,300 g of substrate, 0,090 g of Er^III^-MCM-41 and 30 mL of MilliQ water. For fructose, the reaction was carried out in ten minutes and an internal pressure of 200 psi was recorded in each vessel; for cellulose the reaction time wasf two hours, with an internal pressure of 390 psi.

**Table S1**: *Summary of results gained in the trial for scalability of the process.*

| **Entry** | **Vessel** | **Substrate** | **Conversion^a^** | **Yield^b^ (%)** | | | | **Selectivity** |
| --- | --- | --- | --- | --- | --- | --- | --- | --- |
|  |  |  |  | **Lactic acid** | **5-HMF** | **Furfural** | **Levulinic acid** |  |
| 1 | 1 | Fructose | 90 | 74 | 14 | 0,6 | n.d.^c^ | 83 |
| 2 | 2 |  | 90 | 75 | 15 | 0,7 | n.d. ^c^ | 82 |
| 3 | 3 |  | 87 | 67 | 14 | 0,5 | n.d. ^c^ | 82 |
| 4 | 4 |  | 83 | 61 | 13 | 0,3 | n.d. ^c^ | 82 |
| 5 | 5 |  | 83 | 59 | 11 | 0,4 | n.d. ^c^ | 84 |
| 6 | 6 |  | 89 | 67 | 14 | 0,6 | n.d ^c^ | 82 |
| 7 | 7 |  | 82 | 57 | 12 | 0,3 | n.d. ^c^ | 82 |
| 8 | 8 |  | 86 | 61 | 12 | 0,4 | n.d. ^c^ | 83 |
| 9 | 1 | Cellulose^d^ | 60 | 35 | 2 | n.d.^c^ | n.d.^c^ | 94 |
| 10 | 2 |  | 57 | 29 | 0,8 | n.d. ^c^ | n.d. ^c^ | 97 |
| 11 | 3 |  | 59 | 33 | 1 | n.d. ^c^ | n.d. ^c^ | 96 |
| 12 | 4 |  | 55 | 27 | 0,7 | n.d. ^c^ | n.d. ^c^ | 97 |
| 13 | 5 |  | 53 | 25 | 0,7 | n.d. ^c^ | n.d. ^c^ | 97 |
| 14 | 6 |  | 55 | 28 | 1 | n.d ^c^ | n.d ^c^ | 97 |
| 15 | 7 |  | 58 | 31 | 2 | n.d. ^c^ | n.d. ^c^ | 94 |
| 16 | 8 |  | 53 | 27 | 1 | n.d. ^c^ | n.d. ^c^ | 96 |

1. *Conversion calculated by HPLC-RI analysis*
2. *Yield of products calculated from the analysis of crude reaction with HPLC-UV*
3. *The concentration of the product from HPLC analysis led to yield lower than 0,5 %*
4. *MCC cellulose US pre-treated at 10 W for an hour*

**Characterization of the catalyst after recycles**

Samples were characterized by N2 adsorption/desorption isotherms obtained at the temperature of liquid nitrogen using an automated physisorption instrument (Micromeritics ASAP 2020 analyzer, Peschiera Borromeo, Milano, Italy). The samples were evacuated at 300 °C for 1 h prior to the measurements. Total surface area was calculated according to the BET method. The mesoporous structure of the catalyst has been preserved after the 2^nd^ run, as confirmed by the pattern of idrothermal plot and by the results regarding the pore volume.

**Table S2:** *Characteristics of catalysts surface*

| **Sample** | **BET (mq/g)** | **Pore Volume (cc/g)** | **Pore Diameter (Å)** |
| --- | --- | --- | --- |
| Fresh Catalyst | 625 | 0.45 | 26 |
| Catalyst after 2^nd^ run | 331 | 0.33 | 37 |

**Figure S5:** *Isothermal plot of fresh Er(III) MCM-41*


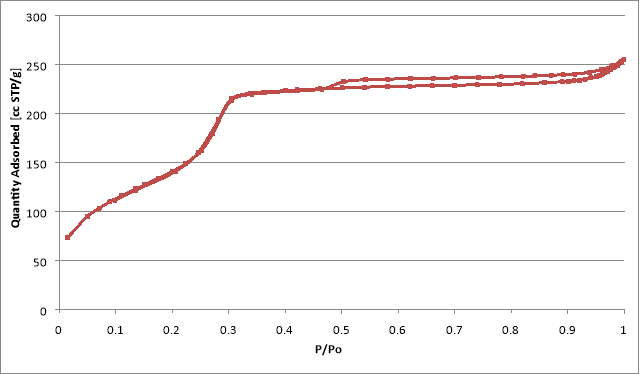


**Figure S6:** *Pore size distribution of fresh Er(III) MCM-41 catalyst*


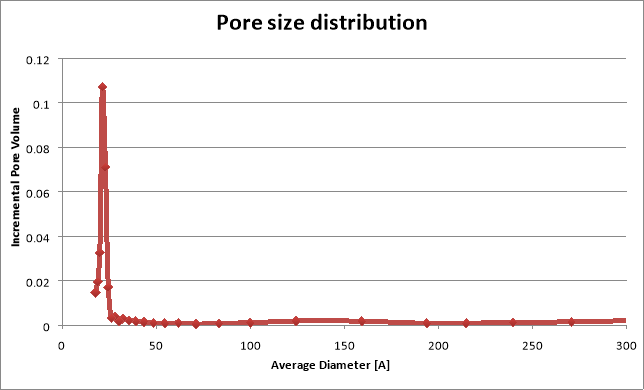


**Figure S7**: *Isothermal plot of Er(III) MCM-41 after 2^nd^ run*


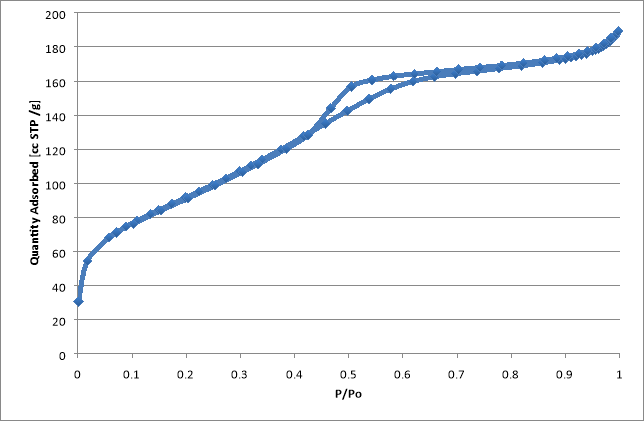


**Figure S8:** *Pore size distribution of Er(III) MCM-41 catalyst after 2^nd^ run*


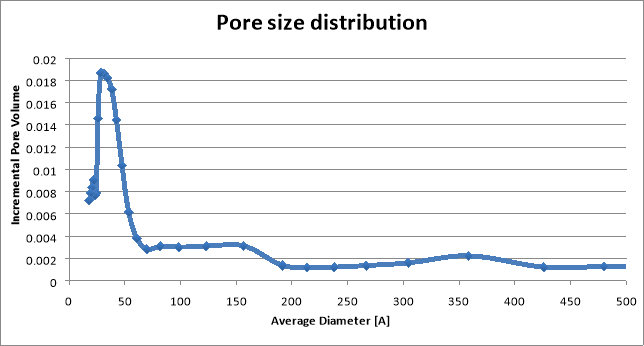


**ICP-MS determination of Er(III) during catalyst recycling**

In order to determine the Er(III) content loaded on the MCM-41 silica surface or leached during reactions, a MW-assisted digestion procedure was applied to the functionalized silica, reaction mix and scrubbing water. Silica loaded with Erbium (~10 mg) or reaction mix and scrubbing water(6 ml) were digested with 8 ml suprapure HNO_3_ (65%, v/v, Merck) using an Anton-Paar Multiwave 3000 microwave digester, equipped with a XF100 rotor (operating pressure: 60 bar). One randomly selected vessel was filled only with reagents and used as a blank. Digestion was conducted in “power controlled” mode. After digestion, the vessels have been cooled down, the digests were filtered with a single use filter unit (0.45µm), then diluted to 50 ml using purity water (obtained from a Milli-Q water purification system, Millipore, France).

ICP-MS measures were performed in a quadrupole-based ICP-MS system XSERIES 2 ICP-MS, from Thermo Fisher Scientific, working in standard mode. Samples were introduced in a quartz concentric nebulizer by a peristaltic pump (selected speed of 30 rpm). Erbium concentration was determined against external calibration using a synthetic acid multielement calibration standard (IV-ICPMS- 71A Inorganic VENTURES).

**Table S3**: *Er(III) content determination during catalyst recycling*.

| **Run of recycle** | | | | | **Heating method** | **% Er(III)** | | | | | | | |
| --- | --- | --- | --- | --- | --- | --- | --- | --- | --- | --- | --- | --- | --- |
|  |  |  | | | | | **Catalyst residue** | | **Reaction mix** | | | **Scrubbing water** | |
|  | 1 | | | MW | | 67 | | 10 | | 1 | | | |
|  | 2 | | | MW | | 4 | | 68 | | 5 | | | |
|  | 2^a^ | | | MW | | 76 | | 24 | | - | | | |
|  | 2^a^ | | conventional | | | 81 | | 19 | | | - | |  |

**a) “Blank” experiment: reaction performed without fructose.**
